# Remote Subthreshold Stimulation Enhances Skin Sensitivity in the Lower Extremity

**DOI:** 10.3389/fnhum.2021.789271

**Published:** 2021-12-22

**Authors:** Emma B. Plater, Vivian S. Seto, Ryan M. Peters, Leah R. Bent

**Affiliations:** ^1^Department of Human Health and Nutritional Sciences, University of Guelph, Guelph, ON, Canada; ^2^Faculty of Kinesiology, University of Calgary, Calgary, AB, Canada

**Keywords:** stochastic resonance, subthreshold noise, vibrotactile, electrotactile, lower limb

## Abstract

Foot sole skin interfaces with the ground and contributes to successful balance. In situations with reduced sensitivity in the glabrous foot skin, stochastic resonance (SR) improves skin sensitivity by adding tactile noise. Some situations, however, involve an interface comprised of hairy skin, which has higher thresholds for sensitivity. For example, in lower extremity amputation the residual limb is comprised of hairy leg skin. The main objective of this study was to determine if SR improves skin sensitivity in hairy skin, and whether a specific intensity of noise is most effective. Secondary objectives were to compare the effect between locations, ages and modalities. In 60 healthy participants a vibrotactile (test) input was delivered at the lower extremity concurrently with a second, noisy stimulus applied more proximally. The presence of a remote SR effect was tested in 15 young participants using electrotactile noise at the calf. Secondary objectives were tested in separate groups of 15 subjects and differed by substituting for one of the three variables: vibrotactile noise, heel site, and with older participants. A forced-choice protocol was used to determine detection ability of the subthreshold vibration test input with varying noise levels applied simultaneously (0, 20, 40, 60, 80, and 100% of perceptual threshold). An SR effect was identified when increased detection of the input was obtained at any level of noise versus no noise. It was found that all four test groups demonstrated evidence of SR: 33–47% of individuals showed better detection of the input with added noise. The SR effect did not appear consistently at any specific noise level for any of the groups, and none of the variables showed a superior ability to evoke SR. Interestingly, in approximately 33% of cases, threshold values fluctuated throughout testing. While this work has provided evidence that SR can enhance the perception of a vibrotactile input in hairy skin, these data suggest that the ability to repeatably show an SR effect relies on maintaining a consistent threshold.

## Introduction

Sensory feedback from the foot sole provides important information for balance control and gait (Zehr and Stein, 1999; Kavounoudias et al., 2001). The glabrous (non-hairy) skin on the foot sole and the cutaneous mechanoreceptors therein are optimized to relay appropriate information for reflexive control of both upper and lower limb muscles (Nurse and Nigg, 2001; Fallon et al., 2005; Bent and Lowrey, 2013; Zehr et al., 2014). There are four classes of receptors within the glabrous skin which relay different types of mechanical input, such as pressure, vibration, slips and skin stretch to the central nervous system (Macefield, 1998). A recent review suggests that 70% of the mechanoreceptors at the foot sole are fast adapting, with the majority being associated with fast-adapting type 1 (FAI) afferents (Strzalkowski et al., 2018). Fast-adapting afferents are of particular importance in the foot, as their sensitivity is related to postural stability in standing (Peters et al., 2016). FAI importance is also highlighted by evidence of strong synaptic coupling of FAI afferents with muscles in the lower extremity (Fallon et al., 2005; Peters et al., 2016, 2020).

Poor balance and falls risk can be related to changes in these receptors in different conditions (Strzalkowski et al., 2018). Advanced age has been associated with a decrease in receptor sensitivity (Perry, 2006; Peters et al., 2016; Mildren et al., 2017), and subsequently poor balance (Peters et al., 2016), possibly due to a decline in the mechanoreceptor population with age (García-Piqueras et al., 2019). Diabetes mellitus results in decreased balance and muscle strength, both of which are worse in the presence of sensory dysfunction and increased age (Kraiwong et al., 2019). Further, older individuals with diabetes mellitus are at risk of requiring lower extremity amputation (LEA) (Imam et al., 2017). Among individuals with LEA who choose to use a prosthesis, the interface between the skin and the support surface is now located where the residuum sits within the prosthetic socket (Li et al., 2015). The base of the residuum is composed of hairy skin from the posterior lower leg, which is not optimized to function as a sensory interface with the ground (Li et al., 2015) due to the reduced number of receptors (Corniani and Saal, 2020). In all of these scenarios a base level of input to the skin needs to be re-established, as it is essential to transduce pressure changes and force distribution to adequately signal weight distribution and therefore reduce the risk of falls (Fan et al., 2008; Strzalkowski et al., 2018). Thus, it is of critical importance to augment the skin sensation that is available.

Stochastic resonance (SR) is a phenomenon already being explored as a means of augmenting skin sensitivity (Collins et al., 1996, 1997; Liu et al., 2002; Priplata et al., 2003; Wells et al., 2005). In cutaneous SR, a weak tactile stimulus is strengthened by the addition of a subthreshold, tactile noise stimulus (Collins et al., 1996, 1997). SR has been shown to improve perception of a tactile stimulus in the glabrous skin of the hand (Collins et al., 1997; Richardson et al., 1998; Kurita et al., 2013; Iliopoulos et al., 2014) and foot (Dhruv et al., 2002; Khaodhiar et al., 2003; Wells et al., 2005). It has also been shown to improve proprioception at the lower extremity (Collins et al., 2009) as well as various measures of balance and gait (Dettmer et al., 2015; Aboutorabi et al., 2017) in healthy individuals, older individuals and those with various sensory conditions.

Questions still remain with respect to the best way to optimize the effectiveness of SR. It is generally accepted that there is an optimal intensity of subthreshold noise that is most effective in enhancing sensitivity (Collins et al., 1996, 1997). Noise is effective because it adds information to the system; if the intensity is too low it will not have the desired effect, but if it is too high it will overtake and distract from the signal of interest (Collins et al., 1997). Research has not been consistent on whether this optimal level is (e.g., Wells et al., 2005) or is not (e.g., Liu et al., 2010) similar between different individuals.

Noise has been shown to be effective in older adults at improving perception (Dhruv et al., 2002; Liu et al., 2002; Breen et al., 2016) and balance measures (Gravelle et al., 2002; Costa et al., 2007; Lipsitz et al., 2015; Zhou et al., 2016; Aboutorabi et al., 2017). Research has shown, in fact, that noise is more effective in the elderly than in the young at improving some postural outcomes (Priplata et al., 2003; Dettmer et al., 2015). Additionally, there is some evidence that even in a population of older adults alone, baseline balance ability is related to the effectiveness of SR, such that those who have poorer balance experience greater improvements with added noise (Stephen et al., 2012). In those who are younger, noise may not be as effective because the sensory system is already optimized; studies have shown that other enhancement techniques such as kinesiology taping and texture are effective only in those with a deficiency that needs to be compensated for Callaghan et al. (2002),Hosp et al. (2015), Lamers et al. (2019). However, only one study has directly compared the effect of SR on perception in younger versus older adults (Wells et al., 2005). This study saw some differences in the optimal frequency of noise in younger versus older adults, but SR appeared to be equally effective at enhancing perception in both age groups (Wells et al., 2005). It is unknown whether age-related differences in SR effectiveness exist in skin regions other than the foot sole.

In fact, while much research has found that SR is effective when applied to glabrous skin (Collins et al., 1997; Dhruv et al., 2002; Priplata et al., 2003), there is a dearth of research on SR effects in hairy skin. As mentioned previously, hairy skin is less optimal as an interface because of its lower receptor density, particularly those of FA afferents (Macefield, 1998; Corniani and Saal, 2020). Considering that sensory augmentation techniques may be more effective when there is a deficiency, hairy skin has the potential to see a greater sensory enhancement from SR compared to glabrous skin. Making a direct comparison between SR effectiveness in glabrous versus hairy skin may help to further understanding of any baseline-dependent effects seen.

One more comparison worth making is that between electrotactile and vibrotactile noise as the modality used to evoke the SR effect. Vibrotactile noise is effective at evoking SR (Liu et al., 2002; Khaodhiar et al., 2003; Cloutier et al., 2009) and targets the mechanoreceptor end organ. Thus, frequency of stimulation can be modified to target specific receptor types, as each responds preferentially to a different range of frequencies (Hao et al., 2015; Strzalkowski et al., 2018). Electrotactile noise is also effective at evoking SR (Dhruv et al., 2002; Breen et al., 2014) but bypasses the end organ and directly influences activity of the neuron (Hao et al., 2015). No studies have directly compared the two modalities; a comparison between the two would not only help inform applications of SR but could also further understanding of the best “route” by which noise is introduced into the system.

The purpose of the current research is to act as proof-of-concept for future application of SR. The work aims to advance knowledge of effective application including benefits for individuals with amputation, and other application to regions with a non-glabrous interface. SR can be applied in two different ways, local and remote. In local SR, the noise is applied to the same skin that is being tested for sensitivity changes; in remote SR, the noise is applied to an area of skin remote from the test area. In LEA, remote SR is ideal to enable noise to enhance skin sensation without a bulky setup interfering with the fit of the residuum in the socket. In the upper extremity, vibrotactile noise at the hand and forearm can enhance sensitivity remotely at the fingertip (Enders et al., 2013). Some support is also provided in the lower limb where noise at the ankle has been shown to improve sensitivity of the glabrous skin of the foot (Breen et al., 2016). While noise has previously been shown to be effective at sensitizing fast-adapting receptors locally (Collins et al., 1997; Dhruv et al., 2002; Wells et al., 2005), little is known about the effects of remote noise in the lower limb on cutaneous receptors.

The primary objectives of this study were to determine whether remote subthreshold electrical stimulation of the hairy skin on the posterior leg improves sensation to vibrotactile input, and whether there is an optimal intensity of noise that is most effective. The secondary objectives were to compare this effect between (1) locations – stimulation at the heel versus the calf (to explore glabrous versus hairy skin), (2) ages – older versus younger individuals, and (3) stimulation modalities – electrotactile versus vibrotactile noise.

## Materials and Methods

Sixty participants were recruited to participate in this study. They were separated based on age (young adults aged 24 ± 4 years, older adults aged 62 ± 5 years). The sixty total participants were divided into four test groups of 15 participants each, described below. All participants indicated no history of a clinically diagnosed condition resulting in sensory loss of the lower extremity and provided informed, written consent to participate in the study. The study was approved by the University of Guelph’s Research Ethics Board.

Overall, SR was tested in this study by assessing participants’ ability to detect a vibrotactile “test” stimulus with and without added tactile noise. The noise was applied at various levels to see if it enhanced the ability to detect the “test” stimulus. Location of the stimuli and modality of the noise differed between groups.

### Objective One: Remote Stochastic Resonance on the Calf With Electrical Noise

**FIGURE 1:**
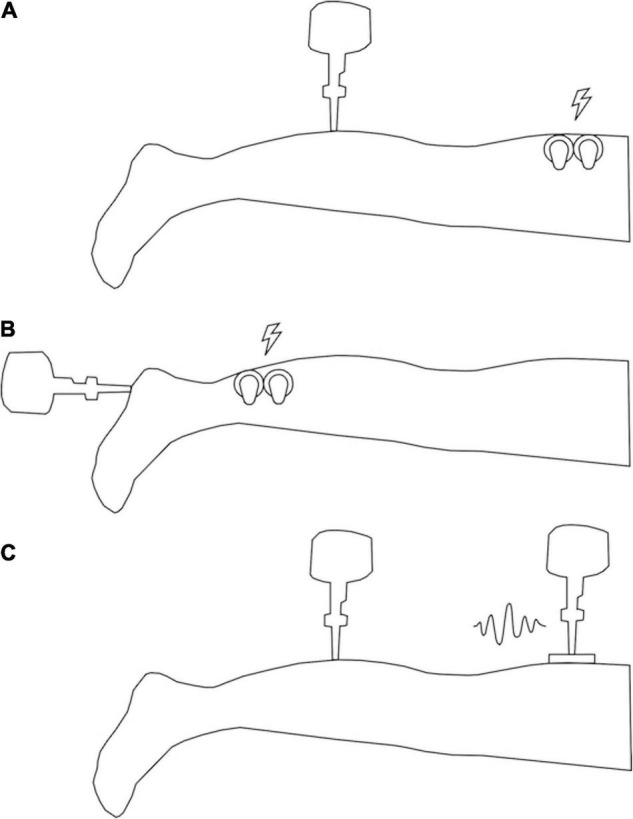
Illustration of participant setup. **(A)** Setup for MAIN and OLD groups (vibrotactile stimulus at the calf and electrotactile noise at the thigh). **(B)** Setup for HEEL group (vibrotactile stimulus at the heel and electrotactile noise at the calf). **(C)** Setup for VIB group (vibrotactile stimulus at the calf and vibrotactile noise at the thigh). Lightning bolts indicate locations of electrotactile noise application and squiggle indicates location of vibrotactile noise application.

Fifteen healthy young participants (7 females, 8 males), with an average age of 24 ± 3 years, an average height of 1.737 ± 0.0773 m and an average weight of 73.5 ± 14.72 kg were recruited as the MAIN testing group. This group experienced the test vibratory input on the calf and electrotactile noise input at the thigh (Figure 1A).

#### Participant Set-Up

Testing was comprised of one session of approximately 90 min. For all testing, participants lay prone on a height-adjustable treatment table with the right (test) limb supported by a VersaForm^®^ pillow to minimize movement. Participants were instructed to lie as still as possible and be attentive to the site being tested. Testing was completed in a quiet room to minimize potential distractions. Short mental breaks were given between parts of the test and on participant request to limit loss of attention.

The following sites on the right limb of each participant were found using bony anatomical landmarks and marked on the skin with pen prior to testing: the test input was applied on the calf, 10 cm below the popliteal crease, and the noisy stimulus was applied 10 cm above the popliteal crease. The calf location was chosen for the test input to mimic the skin surface that is most analogous to the base of the residual limb in transtibial amputees (Li et al., 2015), and to avoid testing over tendons. Hairy skin was shaved to reduce electrical impedance through the skin.

#### Testing Protocol

Each testing session was divided into two parts, perceptual threshold testing (for both test and noise stimuli) followed by testing of the SR effect. Stimulus detection was always tested with a two-interval forced-choice paradigm (2IFC) where two consecutive time points were indicated audibly by beeps. Participants were instructed to indicate verbally if they felt the target stimulus at the first (“first”) or second (“second”) beep.

#### Tactile Input

Vibration at the test site was produced using a probe of 6 mm diameter attached to an electromagnetic vibrator (mini-shaker type 4810, Bruel and Kjaer, Naerum, Denmark), and applied perpendicularly to the skin (see Figure 1A). Vibration frequency was set to 30 Hz to preferentially activate the FAI receptors (Toma and Nakajima, 1995). The frequency was delivered using a custom program in LabVIEW^®^ and a custom-built BNC breakout box and amplified (Power Amplifier Type 2719, Bruel and Kjaer, Naerum, Denmark). Acceleration and force data were collected (acceleration - model 2221D, Endevco, CA, United States; force – Model 31 load cell, Honeywell, MN, United States) at a sampling frequency of 1000 Hz (BNC-2111, National Instruments, Austin, TX, United States). Displacement of the probe was measured by a custom sensor (model RGH24Z, Renishaw, Gloucestershire, United Kingdom) and input *via* a DAQ interface (SCC68, National Instruments, Austin, TX, United States) into the custom LabVIEW^®^ program.

#### Noise Input

Electrotactile non-uniform white noise was applied at the posterior thigh (see Figure 1A) within a frequency band of 0–50 Hz (the band must contain the target frequency of 30 Hz to allow for the resonance effect). Electrical noise was generated with a custom LabVIEW^®^ program and output *via* a DAQ interface (SCC68, National Instruments, Austin, TX, United States) to a constant current isolated stimulator (A395, World Precision Instruments, Sarasota, FL, United States) through two 34 mm round sensor adhesive electrodes (Ambu^®^ BlueSensor M, Ambu Sdn. Bhd., Penanq, Malaysia) affixed to the skin with Transpore^®^ medical tape on either side of the SR location.

#### Part One – Threshold Testing

**FIGURE 2:**
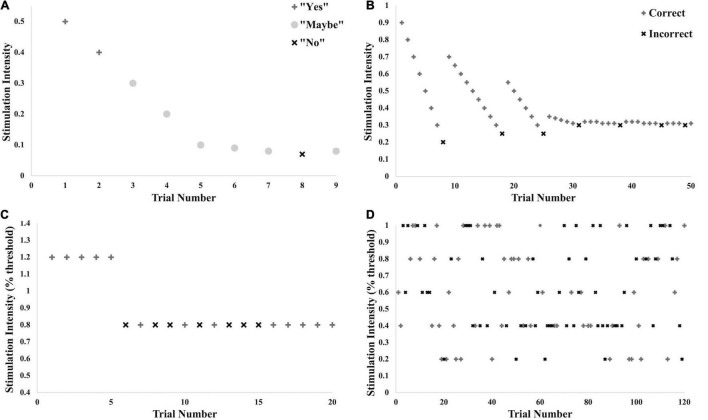
Illustration of testing protocol with example data. These different stages of threshold testing and testing for the SR effect were performed for all trials across all conditions. **(A)** Brief initial threshold testing using a method of adjustment to determine the approximate intensity of the threshold. **(B)** True threshold testing using the limits set in **(A)** and the Bayesian Adaptive Method. **(C)** Practice trials for testing for SR effect: 5 trials above calculated threshold for detection, 15 trials at 80% of threshold. **(D)** Testing of SR effect: 20 trials each at 0, 20, 40, 60, 80, and 100% of threshold for detection.

Testing commenced with detection threshold testing using the method of adjustment, where the stimulus was applied beginning at 0.5 mA and adjusted up or down in intensity until a range was determined within which the threshold fell (see Figure 2A). The second step involved a two-interval forced-choice method of 50 trials using the Bayesian adaptive procedure (Kontsevich and Tyler, 1999; Goldreich et al., 2009) to determine the intensity of each presented stimulus based on previous responses (see Figure 2B). In one of the two stimulus intervals (order randomized), the target stimulus was applied at the intensity determined by the Bayesian adaptive procedure (using expected entropy minimization); in the other stimulus interval no stimulus was applied. The participant had to indicate within which beep the stimulus was applied. Thresholds for both test and noise stimuli were determined (order randomized). Threshold was set at 76% correct where d-prime = 1. Part 1 took approximately 40 min.

#### Part Two – Testing for Stochastic Resonance Effect

The ability to detect the subthreshold stimulation was tested at two consecutive time points indicated audibly by beeps. The vibrotactile stimulus was applied at 80% of the calculated perceptual threshold of the vibrotactile test input (subthreshold), and the electrotactile noise was applied at either 0, 20, 40, 60, 80 or 100% of the determined detection threshold for the noise (control, subthreshold or right at threshold). The different levels of noise were randomized for a total of 20 trials per noise condition. At one of the two time points, both stimuli (test and noise) were applied simultaneously. At the other time point, only the electrotactile noise was applied. The participant was instructed to indicate with which beep the vibrotactile test stimulus was applied.

Testing commenced with 20 practice trials (see Figure 2C), the first five of which supplied a suprathreshold vibration to familiarize the participant with the protocol. The following 15 supplied vibration at 80% of threshold. Noise in all 20 of these trials was supplied at 80% of noise detection threshold. Verbal feedback was given after each trial indicating whether the participant had picked the correct stimulation window. Following practice, 120 trials were given, all with the test vibration at 80% of threshold and noise varying randomly between the six levels (see Figure 2D). No verbal feedback was given at this time. Ten seconds was given between participant response and initiation of the next trial to decrease any lingering sensations at the two sites. Overall, Part 2 took approximately 40 min.

### Secondary Objectives: Location, Age, and Modality Comparisons

To compare the SR effect across locations, ages and modalities, three separate groups of 15 subjects were recruited. For the location comparison, a young group (10 females and 5 males, with an average age of 24 ± 4 years, an average height of 1.723 ± 0.0625 m and an average weight of 67.4 ± 9.91 kg) was tested (“HEEL” group). This comparison examined SR responses between glabrous and hairy skin, in which the test location for the input vibration was the heel (15% of the posterior-to-anterior length of the foot to the base of the toes) and the location of the noise application was the calf (15 cm above the calcaneus) (see Figure 1B). The heel location was chosen to test the SR response because it has been shown previously to evoke an SR effect (Khaodhiar et al., 2003; Priplata et al., 2003; Aboutorabi et al., 2017), which allows for comparison with less sensitive hairy skin. For the age comparison, 15 healthy participants were recruited (7 females and 8 males, with an average age of 62 ± 5 years, an average height of 1.717 ± 0.1072 m and an average weight of 75.5 ± 14.29 kg) (“OLD” group). They underwent the same set up as the control/comparison group, with the test stimulus on the calf and the electrotactile noise on the thigh (see Figure 1A). Finally, for the modality comparison, a young group (10 females and 5 males, with an average age of 23 ± 4 years, an average height of 1.70 ± 0.0959 m and an average weight of 72.9 ± 14.79 kg) was tested with the noise added in a vibrotactile modality (“VIB” group). This vibrotactile noise stimulus was non-uniform white noise within a frequency band of 0–50 Hz. The vibrotactile stimulus was applied initially at 59 ± 21 μm and then adjusted up or down during threshold testing. Vibrotactile noise was applied centrally over the marked location on the heel using a rectangular, plastic probe (10 cm × 5 cm) attached to a second electromagnetic shaker (mini-shaker type 4810, Bruel and Kjaer, Naerum, Denmark), identical to that used for the vibration test input (see Figure 1C). All other aspects of the protocol were the same as for the primary objective.

### Data Processing and Analysis

Data were processed using a custom LabVIEW^®^ program. SR curves were produced with intensity (percent of noise threshold) on the *x*-axis and percent correct (“%correct”) on the *y*-axis. A larger %correct value indicated better detection of the input. Using a binomial test assuming equal likelihood of a correct versus an incorrect response, 67.5% correct is the statistical cutoff corresponding to two standard deviations away from the mean and is generally considered the cutoff above which perception is occurring assuming the baseline %correct is approximately 50% (Collins et al., 1997). However, participants’ %correct with no noise added (0% noise) was more variable than expected and was in many cases even above 67.5% correct. Therefore, to determine whether noise was improving sensitivity in each individual, an SR effect was identified here as any time the %correct value at any level of noise was ≥17.5% (67.5–50%) above that seen at 0% noise.

**FIGURE 3:**
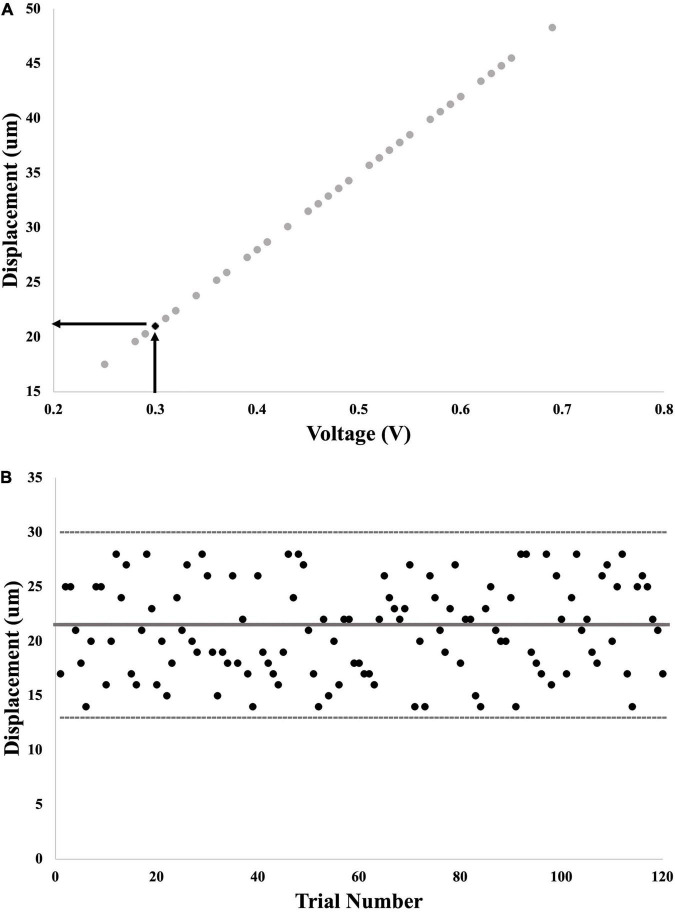
Example calculation of probe displacement from voltage command signal. **(A)** First step: input voltage for each trial in Part 1 was graphed against output displacement of the probe. The final calculated voltage threshold was used to interpolate the matching displacement threshold. **(B)** Second step: output displacement from each of the 120 trials for calculation of SR effect was graphed to determine whether these values fell within 1 standard deviation of displacement threshold (thick gray line = threshold; thin gray lines = 1 standard deviation away).

To confirm that the vibration test input was maintained within 1 standard deviation we matched the calculated test stimulus threshold (in volts – V) to the recorded displacement of the shaker (in microns – μm) (see Figure 3). We first converted the threshold calculated in Part 1 into a displacement value in μm (Figure 3A), and then assessed all of the actual displacement outputs delivered in each trial in Part 2 (Figure 3B). This allowed us to ensure that the actual output of the shaker appropriately matched the input sent from the LabVIEW program.

### Statistical Analysis

Statistics were run using IBM^®^ SPSS^®^ Statistics v25. Normality, homogeneity of variance and sphericity were assessed by Shapiro-Wilk test, Levene’s test and Mauchley’s test, respectively for all variables and comparisons. Outliers were kept within the dataset to maintain statistical power because removing one data point would mean removing an entire participant. Non-parametric tests were used when data were not normally distributed and when transformations were unable to produce normalcy. Where appropriate, Bonferroni-corrected *post hoc* analyses were run on significant interactions. Statistical significance was set at *p* < 0.05.

#### Analysis of Baseline Threshold Data

To determine whether the participants in each test group had significantly different vibrotactile thresholds at the outset of testing, a one-way ANOVA with simple contrasts (comparison group = MAIN) was run with the independent variable being test group (MAIN, HEEL, OLD, and VIB) and the dependent variable being threshold (in μm). Bonferroni-corrected *post hoc* analyses compared the MAIN group to every other group (Table 1).

**TABLE 1:** Columns: participant group, N, demographics (age in years, sex, height in m, and weight in kg).

Group	*N*	Age (y)	Sex (#males)	Height (m)	Weight (kg)	Test Stimulus Threshold (μm)
MAIN	15	24.4 ± 3.22	8	1.737 ± 0.0773	73.5 ± 14.72	10.87 ± 6.30
OLD	15	61.8 ± 5.00*	8	1.717 ± 0.1072	75.5 ± 14.29	27.46 ± 14.56*
HEEL	15	23.5 ± 4.45	5	1.723 ± 0.0625	67.4 ± 9.91	6.120 ± 2.87*
VIB	15	23.1 ± 3.63	5	1.70 ± 0.0959	72.9 ± 14.79	17.46 ± 5.97

* indicates significantly different from the initial group, as per 1-way ANOVA, p < 0.05.

*Test Stimulus Threshold is the average perceptual thresholds for the test stimulus (displacement of the probe in μm; tested at the heel in “HEEL” group and at the calf in all other groups). Data are presented in mean ± standard deviation, n = 60.*

#### Primary Objective

To determine whether SR was generated in the calf, a related-samples Sign test was run with the independent variable being noise level (the participants’ %correct value at 0% noise [“baseline”], versus the participants’ highest %correct value at any other noise level [“optimal”]) and the dependent variable being %correct. To determine whether there is a noise level that is the best at evoking an SR response, a one-way repeated-measures ANOVA was run with the independent variable being noise level (0, 20, 40, 60, 80, and 100% of noise threshold) and the dependent variable being %correct.

#### Secondary Objectives

To determine whether location, age or modality alter the SR effect seen, three separate two-way mixed-measures ANOVAs were run with the between-group independent variable being test group (MAIN versus HEEL, MAIN versus OLD, MAIN versus VIB), the within-group independent variable being noise level (baseline versus optimal), and the dependent variable being %correct. To determine whether a specific noise level is best at evoking an SR response in any of the groups, three separate two-way mixed-measures ANOVAs with simple contrasts (comparison group = 0% of noise threshold) were run with the between-group independent variable being test group (MAIN versus HEEL, MAIN versus OLD, MAIN versus VIB), the within-group independent variable being noise level (0, 20, 40, 60, 80, and 100% of noise threshold), and the dependent variable being %correct.

## Results

### Participant Demographics

Demographic and threshold data are presented in Table 1. Test stimulus threshold was significantly different between groups. *Post hoc* testing revealed significant differences between locations (MAIN versus HEEL) and between ages (MAIN versus OLD). Participants in the MAIN group, with the test at the calf hairy skin, had a significantly higher test threshold than participants in the HEEL group, who received the test stimulus on the glabrous skin [10.87 ± 6.30 μm versus 6.12 ± 2.87 μm, respectively; a difference of 4.75 μm, (95% CI, 0.526 to 8.98), *t*(15) = 2.395, *p* = 0.030]. Participants in the MAIN group, who as a group were younger, had a significantly lower test threshold than participants in the OLD group (10.87 ± 6.30 μm versus 27.46 ± 14.56 μm, respectively, a difference of 16.59 μm, (95% CI, −26.01 to −7.16), *t*(23) = −3.640, *p* = 0.001).

### Voltage Versus Displacement

The average displacement of the shaker consistently fell within 1 standard deviation of the threshold from Part 1 (see Figure 3B). Importantly, due to the ability to confirm the delivered magnitude of the vibration, we are confident that any changes in performance in the added noise conditions is not due to irregular and inconsistent displacement of the shaker.

### Is There a Remote Stochastic Resonance Effect in the MAIN Group?

**FIGURE 4:**
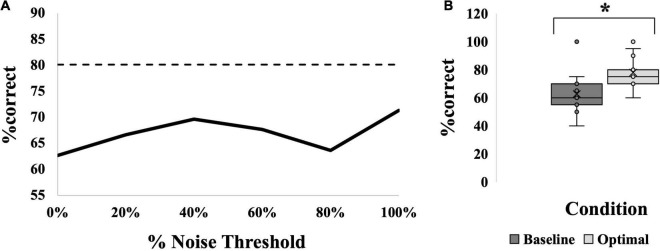
SR effect data for the MAIN group. **(A)** Average SR curve. Each point is the average %correct value for all participants in the group (*N* = 15). Thin dashed line indicates the 17.5% cutoff for an SR effect. **(B)** Box-and-whiskers plot. Baseline (dark gray box; 63%) versus optimal (light gray box; 78%) average %correct values with mean, standard deviation and individual points illustrated. Baseline represents the participants’ %correct value at 0% noise, optimal represents the participant’s highest %correct value at any other noise level. Asterisk indicates significant difference between the bars. Optimal was most frequently seen at 100% noise (6/15 participants).

The presence of an SR effect in the calf occurred in 40% of the participants (6/15); the average SR curve for this group is seen in Figure 4A. This was based on the observation that the %correct was significantly higher at the “optimal” noise level compared to “baseline” (medians = 60% versus 75%, *p* < 0.001; Figure 4B). Optimal was most frequently seen at 100% (6/15 participants). However, the SR effect was not significantly greater at any one noise level compared to the others, *F*(5,70) = 1.965, *p* = 0.095, partial eta^2^ = 0.123. The average SR curve (Figure 4A) indicates that there was a trend toward an effect at 40% but this was not significant.

### Do Location, Age and/or Modality Alter the Effectiveness of Stochastic Resonance?

#### Location Comparison

**FIGURE 5:**
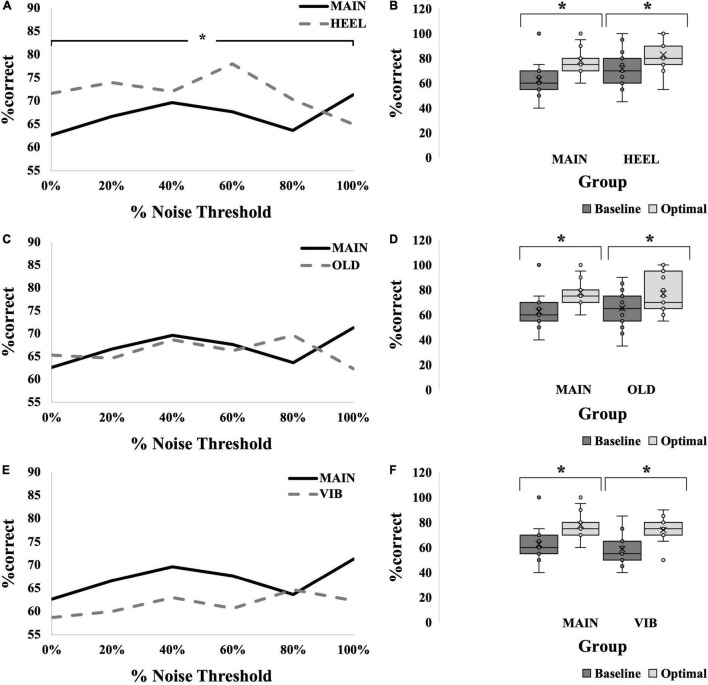
SR effect data for all groups. **(A,C,E)** Average SR curves for calf, young, electrotactile MAIN group (solid black line) versus heel (thick dashed gray line) **(A)**, versus old (thick dashed gray line) **(C)**, and versus vibrotactile (thick dashed gray line) **(E)** comparisons. Each point is the average %correct value for all participants in the group (*N* = 15 for each group). **(B,D,F)** Box-and-whiskers plots. Baseline (dark gray boxes) versus optimal (light gray boxes) average %correct values, with means, standard deviations and individual points illustrated, for calf versus heel **(B)**, young versus old **(D)** and electrotactile versus vibrotactile **(F)** comparisons. Baseline represents the participants’ %correct value at 0% noise, optimal represents the participant’s highest %correct value at any other noise level. Optimal %correct for the calf group and heel group were 78 and 83, respectively; for the young group and old group were 78 and 77, respectively; and for the electrotactile group and vibrotactile group were 78 and 74, respectively. Asterisks indicate significant difference between the bars.

**FIGURE 6:**
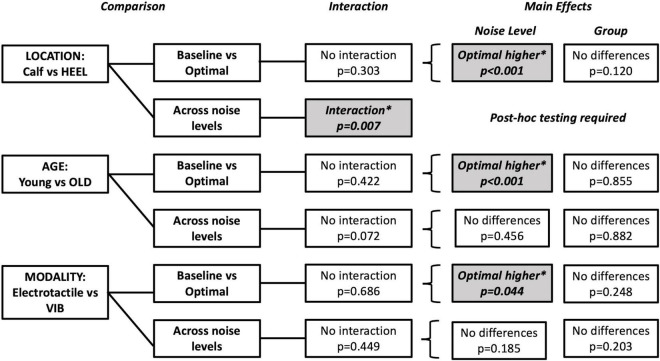
Summary of tests performed and significant findings for the secondary objectives, comparisons between locations, ages and modalities. Significance is *p* < 0.05. Baseline versus Optimal compared the %correct at baseline (0% noise) to the %correct at the optimal noise level (the level with the largest %correct value). Comparisons across noise levels compared no noise (0%) to all other levels (20, 40, 60, 80, and 100%). Shaded boxes with asterisks indicate significant findings.

An SR effect was elicited in 33% of participants in the HEEL group (5/15), which was one participant less than the MAIN group. The average SR curve for the HEEL group is seen in Figure 5A. A summary of statistical findings is seen in Figure 6.

##### Baseline Versus Optimal

Optimal was most frequently seen at 60% for the HEEL group (8/15 participants). There was no statistically significant interaction between group (MAIN versus HEEL) and noise level (baseline versus optimal) on %correct, *F*(1,28) = 1.103, *p* = 0.303, partial eta^2^ = 0.038. The main effect of noise level showed that the %correct was significantly higher at the “optimal” (highest) noise level compared to “baseline,” *F*(1,28) = 49.015, *p* < 0.001, partial eta^2^ = 0.636 (Figure 5B). There were no significant differences between groups, *F*(1,28) = 0.120, *p* = 0.120, partial eta^2^ = 0.084, indicating that there were no differences at the calf versus the heel.

##### Across Noise Levels

There was a statistically significant interaction between group (MAIN versus HEEL) and noise level (0, 20, 40, 60, 80, and 100%) on %correct, *F*(5,140) = 3.307, *p* = 0.007, partial eta^2^ = 0.064. *Post hoc* tests revealed significant differences between noise levels at the heel (*p* = 0.011) but no significant differences between noise levels at the calf (*p* = 0.095). At the heel, %correct was significantly greater at 0% compared to 100% (71.67 ± 14.960 versus 65.00 ± 19.180, *p* = 0.027), at 20% compared to 100% (74.00 ± 14.417 versus 65.00 ± 19.180, *p* = 0.006), at 60% compared to 100% (78.00 ± 17.300 versus 65.00 ± 19.180, *p* = 0.031), and at 60% compared to 80% (78.00 ± 17.300 versus 70.33 ± 19.223, *p* = 0.002) (Figures 5A, 6 and Table 2).

**TABLE 2:** *Post hoc* comparisons following the two-way mixed-measures ANOVA for (HEEL versus MAIN x 0, 20, 40, 60, 80, and 100% noise).

Noise level 1 (%)	%correct	Noise level 2 (%)	%correct	Sig (p)
0	71.67 ± 14.960	20	74.00 ± 14.417	0.324
		40	72.07 ± 19.081	0.914
		60	78.00 ± 17.300	0.109
		80	70.33 ± 19.223	0.698
		100	65.00 ± 19.180	0.027*
20	74.00 ± 14.417	40	72.07 ± 19.081	0.620
		60	78.00 ± 17.300	0.222
		80	70.33 ± 19.223	0.334
		100	65.00 ± 19.180	0.006*
40	72.07 ± 19.081	60	78.00 ± 17.300	0.149
		80	70.33 ± 19.223	0.512
		100	65.00 ± 19.180	0.097
60	78.00 ± 17.300	80	70.33 ± 19.223	0.002*
		100	65.00 ± 19.180	0.031*
80	70.33 ± 19.223	100	65.00 ± 19.180	0.171

* indicates significantly different from the initial group, as per t-test, p < 0.05.

*The %correct value at each noise level was compared to each other noise level, with significant differences indicated by an asterisk. All of these data are from the HEEL group; the MAIN group did not yield any significant differences. Data are presented in mean ± standard deviation.*

#### Age Comparison

An SR effect was elicited in 33% of participants in the OLD group (5/15), which was one participant less than the MAIN group. The average SR curve for the OLD group is seen in Figure 5C. A summary of statistical findings is seen in Figure 6.

##### Baseline Versus Optimal

Optimal was most frequently seen at 80% for the OLD group (7/15 participants). There was no statistically significant interaction between group (MAIN versus OLD) and noise level (baseline versus optimal) on %correct, [*F*(1,28) = 0.663, *p* = 0.422, partial eta^2^ = 0.023]. The main effect of noise level showed that the %correct was significantly higher at the “optimal” noise level compared to “baseline,” *F*(1,28) = 35.965, *p* < 0.001, partial eta^2^ = 0.562 (Figure 5D). There were no significant differences between groups, *F*(1,28) = 0.034, *p* = 0.855, partial eta^2^ = 0.001.

##### Across Noise Levels

There was no statistically significant interaction between group (MAIN versus OLD) and noise level (0, 20, 40, 60, 80, 100%) on %correct, *F*(5,140) = 2.076, *p* = 0.072, partial eta^2^ = 0.069. There were no significant main effects of noise levels (*F*(5,140) = 0.941, *p* = 0.456, partial eta^2^ = 0.033) or groups (*F*(1,28) = 0.023, *p* = 0.882, partial eta^2^ = 0.001) (Figures 5C, 6).

#### Modality Comparison

An SR effect was elicited in 47% of participants in the VIB group (7/15), which was one participant greater than the MAIN group. The average SR curve for the VIB group is seen in Figure 5E. A summary of statistical findings is seen in Figure 6.

##### Baseline Versus Optimal

Optimal was most frequently seen at both 80 and 100% for the VIB group (5/15 participants each). There was no statistically significant interaction between group (MAIN versus VIB) and noise level (baseline versus optimal) on %correct, *F*(1,28) = 0.167, *p* = 0.686, partial eta^2^ = 0.006. The main effect of noise level showed that the %correct was significantly higher at the “optimal” noise level compared to “baseline,” (*F*(1,28) = 4.433, *p* = 0.044, partial eta^2^ = 0.137) (Figure 5F). There were no significant differences between groups, (*F*(1,28) = 1.389, *p* = 0.248, partial eta^2^ = 0.047).

##### Across Noise Levels

There was no statistically significant interaction between group (MAIN versus VIB) and noise level (0, 20, 40, 60, 80, and 100%) on %correct, *F*(5,140) = 0.954, *p* = 0.449, partial eta^2^ = 0.033. There were no significant main effects of noise levels (*F*(5,140) = 1.529, *p* = 0.185, partial eta^2^ = 0.052) or groups (*F*(1,28) = 1.703, *p* = 0.203, partial eta^2^ = 0.057) (Figures 5E, 6).

**FIGURE 7:**
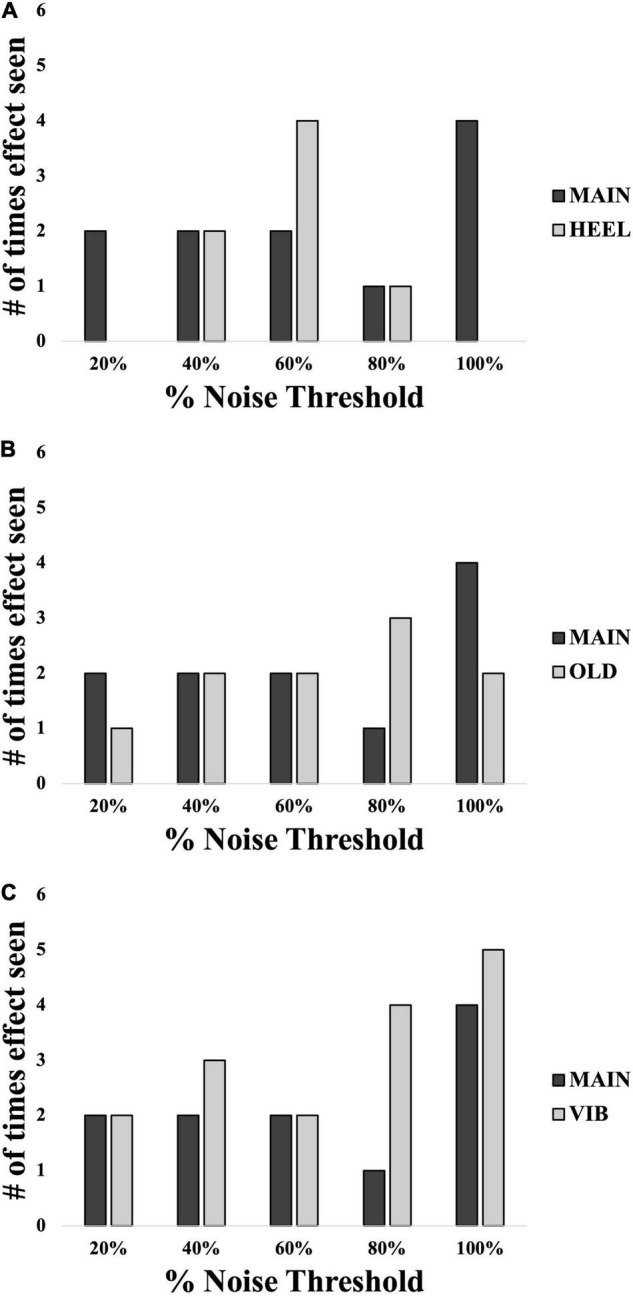
Number of participants in each group showing an SR effect at each% noise threshold level: comparisons between location **(A)**, age **(B)** and modality **(C)**. Black bars represent the MAIN group with calf, young and electrotactile variables. Light gray bars represent the other groups as indicated. *N* = 60 total.

In all comparisons, across all different noise levels no group appeared to have an optimal level of noise that most effectively evoked an SR effect (Figure 7). An overall effect size of f(U) = 0.357 was seen with an associated power of 0.91. In many instances here, the “baseline “%correct value was above 67.5% (MAIN group = 5/15 participants, HEEL = 9/15, OLD = 6/15, VIB = 3/15), the conventional level considered to be baseline threshold.

### Exploratory Analysis: Collapsing Across Groups

**FIGURE 8:**
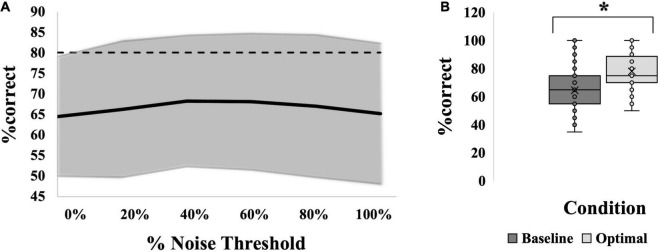
SR effect data for all 60 participants averaged. **(A)** Average SR curve. Each point is the average %correct value for all participants (*N* = 60). Thin dashed line indicates the 17.5% cutoff for an SR effect. **(B)** Box-and-whiskers plot. Baseline (dark gray box; 65%) versus optimal (light gray box; 78%) average %correct values with mean, standard deviation and individual points illustrated. Baseline represents the participants’ %correct value at 0% noise; Optimal represents the participant’s highest %correct value at any other noise level. Asterisk indicates significant difference between the bars. Optimal was most frequently seen at 80% noise (22/60 participants).

Most group comparisons were found to be insignificant. As a result, an exploratory analysis was performed collapsing across groups so that 60 individuals were pooled across location, age and modality. When all groups were averaged together, the resultant curve can be seen in Figure 8A. Here, *via* independent samples t-test, “optimal” noise was significantly higher than “baseline” (64.58 ± 14.6 versus 78.00 ± 12.2, a difference of 13.42%, (95% CI, −16.513 to −10.321), *t*(59) = −8.671, *p* < 0.001; Figure 8B, showing a very robust SR effect across the 60 individuals. However, *via* 1-way repeated-measures ANOVA, no noise level produced significantly higher %correct than “baseline,” which is the %correct at 0% noise, *F*(5,315) = 1.563, *p* = 0.170 (Figure 8A).

## Discussion

We set out to investigate whether remote subthreshold noise at the hairy skin on the posterior leg improves sensation to vibrotactile input, and whether there is an optimal intensity of noise that is most effective. We found that the SR effect was shown in the calf of healthy subjects, providing the first evidence of SR at a hairy skin location. Furthermore, although a significant SR effect was generated for each group (age, modality, and location), we did not find a consistent optimal level of noise that most effectively evokes SR for any test condition.

### Capturing the Stochastic Resonance Effect

Previous studies have analyzed the SR effect in one of three different ways: either comparing performance without noise to (1) one non-zero noise level (Liu et al., 2002; Khaodhiar et al., 2003; Cloutier et al., 2009), (2) a previously determined most effective/”optimal” level (Liu et al., 2010; Breen et al., 2014, 2016) or (3) comparing across various levels of noise (Wells et al., 2005; Enders et al., 2013; Kurita et al., 2013; Li et al., 2015)5. In the current study, when comparing “baseline” to “optimal,” “optimal” noise yielded greater performance than “baseline,” as was seen in previous studies (Liu et al., 2010; Breen et al., 2014, 2016).

However, when comparing the performance at each noise level to that of “baseline,” there were no significant differences in performance between different noise levels. Lack of statistical significance for the comparison across different levels of noise can be explained by the large amount of variability in the data. This finding is consistent with previous SR work that has also seen variability, both in measured neural responses (Manjarrez et al., 2002, 2003) and in perception (Liu et al., 2010). Manjarrez et al. (2002, 2003) suggested many possible physiological causes for variability, including variability in skin structure and elasticity, density of end receptors and multi-level “background firing activity” in the nervous system (Manjarrez et al., 2002, 2003). For instance, there is a background level of electrical noise (i.e., random, spontaneous neuronal firing) occurring at the synapses of all levels of the central nervous system, including the spinal cord, brainstem, thalamus and cerebral cortex (Manjarrez et al., 2003). Therefore, the total amount of activity that reaches and is processed by the cortex is likely variable across even a short time and with every percept. One study that did find significant differences between noise levels also reported very low levels of variability (Wells et al., 2005).

### Location Comparison

Baseline test stimulus threshold values were significantly different between locations, with the mean threshold in the MAIN group, with the test at the calf, being almost double that of the threshold of the HEEL group (MAIN = 10.87 μm versus HEEL = 6.12 μm). Despite having very sensitive fast-adapting afferents, the hairy skin has a greater proportion of slow-adapting afferents and fewer FA receptors may have been activated by our stimulus (Macefield, 1998). A recent study found an innervation density of 16 afferents/cm^2^ at the heel, with 37% being SA, while generally the innervation density at the leg was much less (9 afferents/cm^2^) with a much greater proportion (65%) being SA (Corniani and Saal, 2020). As such it was not surprising to find significantly higher baseline thresholds in the hairy skin.

An SR effect, where noise enhanced the ability to perceive the test stimulus, was seen in a subset of individuals in each group, when either the calf or the heel were test sites. The significant interaction for the comparison between different noise levels (0% greater than 20, 60, and 100%, 60% greater than 80%; see Table 2) indicates that the heel (glabrous skin) responded differently than the calf (hairy skin) across the different noise levels. For our purposes, we were only interested in differences between 0% noise and other non-zero noise levels. Our finding that %correct at 0% noise was greater than at 100% noise (see gray dashed line in Figure 5A) was the opposite from what was expected, and suggests performance was better in the absence of noise here than in the presence of it. Despite efforts to effectively assess perceptual threshold and deliver a test stimulation that was 80% of this, the %correct obtained with no noise indicated that the stimulus was above threshold for over half of the participants in the HEEL. As mentioned earlier, the ability to correctly identify vibration at 0% noise should be approximately 50% correct, and no greater than 67.5% correct, corresponding to random chance if the vibration was below threshold. Values above 67.5% correct suggest that the vibration was suprathreshold. In the HEEL group, 9 of 15 participants had a %correct value above 67.5% at 0% noise (75, 80, 100, 75, 95, 85, 70, 80, and 70% correct). It is generally believed that suprathreshold noise intensity is detrimental to signal detection because, at this high level of the parabola, the noise now overpowers the signal and no longer enhances it (Collins et al., 1997). In this context the result makes sense: perceptual performance at 100% noise was likely worse than that at 0% because the suprathreshold noise level was overpowering the signal.

### Age Comparison

As expected, baseline test stimulus thresholds were significantly different between ages, with the mean threshold in the older group being almost three times that of the younger group (OLD = 27.46 μm versus MAIN = 10.87 μm). At the foot sole, Wells et al. saw thresholds of approximately 50 μm for FAI receptors (at 25 Hz) for both older adults and younger adults, with no significant differences between ages (Wells et al., 2003). In our current study with similar age ranges to Wells, there were significant differences between younger and older participants’ thresholds. Perhaps the location of the test stimulus, the calf, is what made the difference here. Skin mechanical properties such as hardness and thickness vary between body regions (Smalls et al., 2006). Thinner and softer skin generally has lower sensitivity thresholds (Strzalkowski et al., 2015). However, at the foot sole, VPT does not correlate with skin hardness and thickness, possibly because all skin at the foot sole is relatively hard and thick (Strzalkowski et al., 2015). Perhaps the differences in mechanical properties, at the calf, between older and younger individuals is greater, and so differences in VPT may be amplified as well.

There were no significant differences in the SR effect between older and younger individuals in this study, demonstrating the capacity for SR to have potential in this population. Only one previous study directly compared perceptual SR effects in healthy younger adults to healthy older adults, with no differences in relative performance between the two age groups as was seen in our study (Wells et al., 2005). They tested 12 participants and six noise levels: 0, 33, 50, 67, 83, and 100% threshold (Wells et al., 2005). In the Wells study, when the test stimulus was set to 80% threshold and FAI-mediated frequencies were used, the optimal noise level in both older and younger individuals were consistently found to be 50% threshold (Wells et al., 2005). The current study did not find one optimal noise level, but also did not include 50% noise threshold, so the optimal range could have been missed if it is small. However, notably, we did not observe a trend toward optimal at our 40% or 60% noise levels; we suspect it is more likely that we did not see an optimal level due to variability in our data. Also, local noise was utilized in the Wells study, with the vibrotactile stimulus and noise being applied simultaneously *via* the same probe (Wells et al., 2005), while the current study utilized remote noise. The differences between local and remote SR are discussed in a later section.

### Modality Comparison

Both vibrotactile and electrotactile noise produced an SR effect in different individuals in the current study, and individual optimal intensities varied between individuals. Mechanistically, it is believed that vibrotactile noise sensitizes the system by adding energy to the signal directly at the sensory end organ, making the signal stronger (Hao et al., 2015). The effects of electrotactile noise are thought to be indirect, bypassing the end organ and contributing instead to the afferent gain system, which is the amount of information traveling up the afferent nerve to the spinal cord (Hao et al., 2015). Of the studies looking at vibration perception, similar amounts of improvement in perceptual threshold were seen with vibrotactile noise [30% (Liu et al., 2002), 26% (Khaodhiar et al., 2003), 18% (Cloutier et al., 2009) improvement], and electrotactile noise [16% (Breen et al., 2014) improvement]. From our work we are in agreement that both modalities are effective in generating an SR response to a tactile perception.

### Collapsing Across Groups

When examining the data across all 60 participants, there is no single noise level that is significantly better at producing an SR effect than any other. This comparison was undertaken to determine if an optimal level of noise could be seen with a larger sample size, but again substantial variability was observed here that precluded any single noise level from reaching statistical significance. As discussed above, a previous study found 50% noise to be optimal for improvements in perception; perhaps 50% noise would have reached significance if it were tested in the current study (Wells et al., 2005). Other studies have found 75% (Kurita et al., 2013) or 90% (Cloutier et al., 2009) noise to be effective across their study populations, which were also not levels tested in the current study, so the most effective level may simply have been missed in our study population.

### Importance and Effectiveness of Remote Stochastic Resonance

The choice of the main stimulation locations for this study (test stimulus on the calf and noise stimulus on the thigh) assumes a situation where the noise must be applied proximal to the base of a residual limb in LEA. Remote SR could be helpful in situations such as this where it would be detrimental to apply noise to the same site where sensation needs to be enhanced (Enders et al., 2013). It would additionally be helpful in medical conditions where sensory loss is seen distally but proximal skin sensitivity is intact (Breen et al., 2016).

The mechanism for remote SR is not known, but it is speculated that noise increases excitability of the central nervous system (i.e., at or above the level of the spinal cord) as a whole or in regions that have interneuronal connections (e.g., dermatomes) (Enders et al., 2013; Breen et al., 2014). SR may also improve synchrony of firing between neurons at the spinal cord and neurons at the cortex, enhancing transmission of information from one of these places to the other (Enders et al., 2013; Breen et al., 2014). In our study, for testing at the calf in the MAIN group, the neural connections would be within the pathway from first-order afferents originating at mechanoreceptors at the calf to their axons traveling up the posterior thigh. These axons would be anatomically situated to be influenced by noisy stimulation at the thigh because both locations are within the S2 dermatome (Lee et al., 2008). Similarly, for testing at the heel in the HEEL group, both the location of the stimulation at the heel and noise at the calf are found within the S1 dermatomal region (Lee et al., 2008).

Khaodhiar et al. (2003) directly compared local stimulation to remote (Khaodhiar et al., 2003). Light touch detection was enhanced locally (the foot sole) but not remotely (the great toe) with vibrotactile noise applied to the foot sole (Khaodhiar et al., 2003). However, noise did enhance vibration sensitivity remotely at the great toe (Khaodhiar et al., 2003). Therefore, it is possible that some sensory modalities (such as vibration, as used in the current study) can be much more readily enhanced by remote SR than others (such as light touch).

### Limitations

Perceptual testing is associated with some inherent subjectivity. It is commonly believed that a perceptual threshold is not a single entity; rather, it varies within a range based on several factors including participant expectations and biases (Green and Swets, 1966). For example, if an air traffic controller during wartime is asked to determine whether small objects in the distance are enemy aircraft or birds, they are more likely to label them as enemy aircraft if they are unsure because of the higher risk involved in a “miss” than a “false positive” (Green and Swets, 1966). If there is a greater perceived risk or perceived reward with detection or non-detection, the threshold will be shifted one way or the other (Green and Swets, 1966). Additionally, attention can play a role in whether or not detection occurs; a stimulus very close to “true threshold” may not be detected if the participant is not paying close attention (Green and Swets, 1966).

During pilot testing in this study, we initially used a modified Method of Limits to determine threshold. The given stimulus started above the participant’s threshold and incrementally decreased until the participant reported that they did not feel it. Then, the stimulus was increased from a subthreshold level until the participant stated that they were able to feel it. These “runs” were repeated a set number of times and the final value was taken as threshold. Using this technique, we noticed that in Part 2 (Testing of SR Effect) several participants (∼33%) scored above threshold in the 0% noise condition, indicating that they could now perceive the stimulus that we had previously calculated to be subthreshold.

Given that Methods of Limits are subjective and inherently susceptible to participant biases, criteria and attentional differences (Green and Swets, 1966), we tested several modifications meant to reduce the subjectivity of the task. First, we moved to the more rigorous Bayesian Adaptive Procedure described above. In conjunction, we also implemented the two-interval forced-choice task that greatly reduces the effects of bias and expectation compared to one-interval tasks. However, the thresholds continued to be overestimated with the same frequency in this protocol. We also tried using a test stimulus intensity of 90% of the estimated threshold for Part 2, then reduced it to 80%, and then further reduced it to 60%. Each time, the participants whose thresholds were overestimated continued to score higher than chance in their %correct. For the final protocol, 80% of the estimated threshold was used. It is also worth noting that it was not possible to determine if threshold drifted in the other direction – we could only see if a previously established subthreshold stimulus became suprathreshold. Additionally, we could not determine whether our noise stimulus threshold drifted throughout the protocol. As a result, the ability to produce an SR effect and find a single optimal level of noise may have been influenced.

What appears to be occurring is a drift in perceptual performance throughout the testing session. Thresholds are not fixed; they vary depending on many factors, especially attention. While we attempted to maximize our study’s rigor by taking measures to minimize subjectivity, it is apparent that attention and other factors still played a role in the fluctuation of threshold values over time. Time is a considerable factor in the accuracy of perceptual thresholds. This study took over an hour to complete, which likely influenced the ability of the participants to concentrate and stay appropriately motivated throughout. For optimal accuracy of perceptual testing, there may need to be a balance between study length and the level of subjectivity in the measure itself.

For the purposes of this study, we chose to pool “subthreshold” and “suprathreshold” resultant curves together and label the presence of an SR effect anything that was >17.5% correct greater than at baseline. This way, the %correct was compared to each individual’s starting value at baseline, as opposed to one set value that would not take suprathreshold starting values into account. The 17.5% cutoff could then indicate improved perception even if the baseline was suprathreshold.

Additionally, it should be noted that a different group of individuals comprised each group, and no comparisons (i.e., vibration versus electrical noise, calf versus heel) were made in a single participant. Thus, our location and modality comparisons are considered across a population, and we cannot be sure if a single individual would have differences between these conditions.

## Conclusion

This study provides evidence that remote noise can enhance the perception of a vibrotactile input at the hairy skin of the calf. However, there does not appear to be a consistent level of noise that can best evoke the SR effect during a subjective perceptual threshold task, nor do there appear to be age, location or modality specific differences. Notably, there are limitations of perceptual testing: attention and other cognitive factors that can cause threshold to drift over a relatively brief amount of time. Results of perceptual SR testing rely heavily on the accurate determination and maintenance of a near, but subthreshold, signal. Further work will need to be undertaken to compare various methodologies and determine the best way to accurately determine and maintain perceptual threshold levels.

## Data Availability Statement

The raw data supporting the conclusions of this article will be made available by the corresponding author upon reasonable request.

## Ethics Statement

The studies involving human participants were reviewed and approved by Research Ethics Board, University of Guelph. The patients/participants provided their written informed consent to participate in this study.

## Author Contributions

EP, VS, RP, and LB contributed to the conception and design of the study. EP and VS collected the data for the study. EP performed the statistical analysis and wrote the first draft of the manuscript. EP, RP, and LB contributed to the data interpretation. All authors contributed to the manuscript revision, read, and approved the submitted version.

## Conflict of Interest

The authors declare that the research was conducted in the absence of any commercial or financial relationships that could be construed as a potential conflict of interest.

## Publisher’s Note

All claims expressed in this article are solely those of the authors and do not necessarily represent those of their affiliated organizations, or those of the publisher, the editors and the reviewers. Any product that may be evaluated in this article, or claim that may be made by its manufacturer, is not guaranteed or endorsed by the publisher.
